# Genetic Code Engineering by Natural and Unnatural Base Pair Systems for the Site-Specific Incorporation of Non-Standard Amino Acids Into Proteins

**DOI:** 10.3389/fmolb.2022.851646

**Published:** 2022-05-24

**Authors:** Michiko Kimoto, Ichiro Hirao

**Affiliations:** Institute of Bioengineering and Bioimaging (IBB), Agency for Science, Technology and Research (A^*^STAR), Singapore, Singapore

**Keywords:** translation, genetic alphabet expansion, unnatural amino acid, genetic code expansion, unnatural base pair

## Abstract

Amino acid sequences of proteins are encoded in nucleic acids composed of four letters, A, G, C, and T(U). However, this four-letter alphabet coding system limits further functionalities of proteins by the twenty letters of amino acids. If we expand the genetic code or develop alternative codes, we could create novel biological systems and biotechnologies by the site-specific incorporation of non-standard amino acids (or unnatural amino acids, unAAs) into proteins. To this end, new codons and their complementary anticodons are required for unAAs. In this review, we introduce the current status of methods to incorporate new amino acids into proteins by *in vitro* and *in vivo* translation systems, by focusing on the creation of new codon-anticodon interactions, including unnatural base pair systems for genetic alphabet expansion.

## Introduction

**FIGURE 1:**
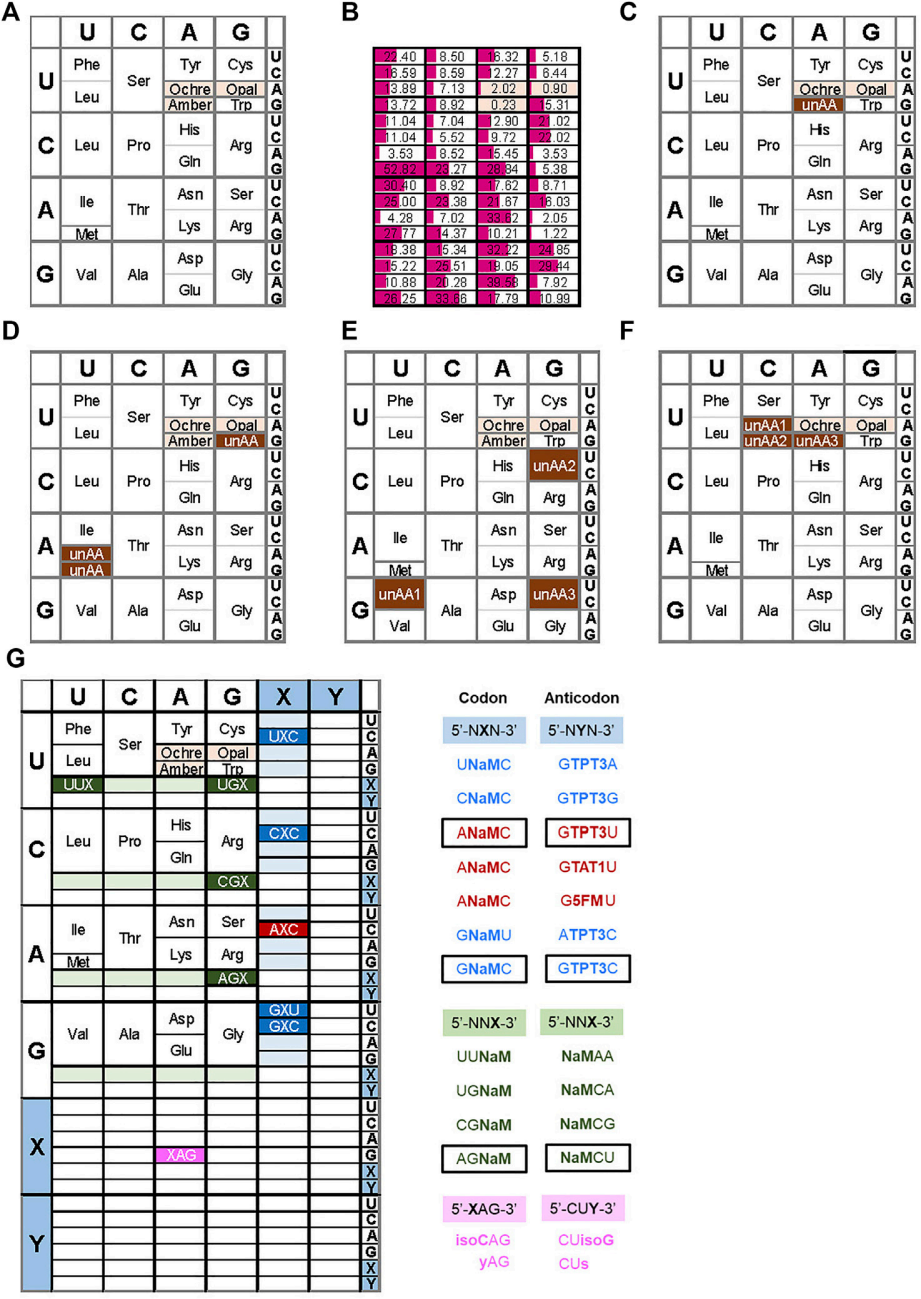
Examples of expanded genetic codon tables **(A)** The original genetic codon table **(B)** Relative frequencies of codon usage in *E. coli* **(C)** Reassignment of the amber codon to an unnatural amino acid (unAA) **(D)** Use of sense codons for unAA, such as Trp codon (UGG) for an unAA (i.e., 4-fluorotryptophan) **(E)** Example of the reprogrammed genetic codon table by Suga’s RAPID method **(F)** Reprogrammed genetic codon table in the engineered *E. coli* (Syn61) **(G)** Expanded genetic codon table using UBPs. Examples of the reported UB codon-anticodons are shown on the right.

The genetic code on earth is ruled by the combinations of three consecutive base sequences as codons corresponding to each amino acid to construct proteins. Sixty-four codons composed of four letters, A, G, C, and T(U), are assigned to the twenty letters of standard amino acids and the three termination signals (stop codons) in translation (Figure 1A). Living organisms maintain the integrity of nucleic acids and proteins within the constraints of the four natural bases and twenty standard amino acids, respectively, by the evolutionary equilibrium between precise information flow through the cognate base pairings, A–T and G–C, and mutations through non-cognate mispairings. However, the limited chemical and biological diversity of these canonical components restricts further improvement toward the development of increased functionalities of nucleic acids and proteins and their biosystems. In fact, living organisms use a wide variety of modified nucleotides and non-standard amino acids (Ambrogelly et al., 2007). For example, d-amino acids, instead of the standard l-amino acids, often appear in peptides and proteins (Heck et al., 1994; Kreil, 1994; Kreil, 1997). Modified nucleotides produced by posttranscriptional modifications of tRNAs increase the efficiency and fidelity of the near cognate codon-anticodon interactions (Agris et al., 2007; Vare et al., 2017; Koh and Sarin, 2018). Therefore, artificially introducing unnatural bases (UBs) and unnatural amino acids (unAAs) into nucleic acids and proteins could increase their functionalities by expanding the genetic alphabet, and thus lead to the creation of newly engineered organisms.

The artificial incorporation of unAAs into proteins through the natural base pair (NBP) or unnatural base pair (UBP) systems requires an orthogonal “bypassing” system for the pre-existing genetic information flow in the central dogma: replication, transcription, and translation. Living organisms have evolved mechanisms to avoid the misincorporation (non-cognate) events of UBs and unAAs and remove these extra components as errors during nucleic acid and protein biosynthesis. Accordingly, in nature, most of the unnatural components in biopolymers are introduced by post-biosynthesis modifications or other biosynthetic mechanisms. To circumvent the proofreading systems of living organisms, researchers have created several “bypassing” schemes by modifying the genetic information flow systems, including the codon table.

**FIGURE 2:**
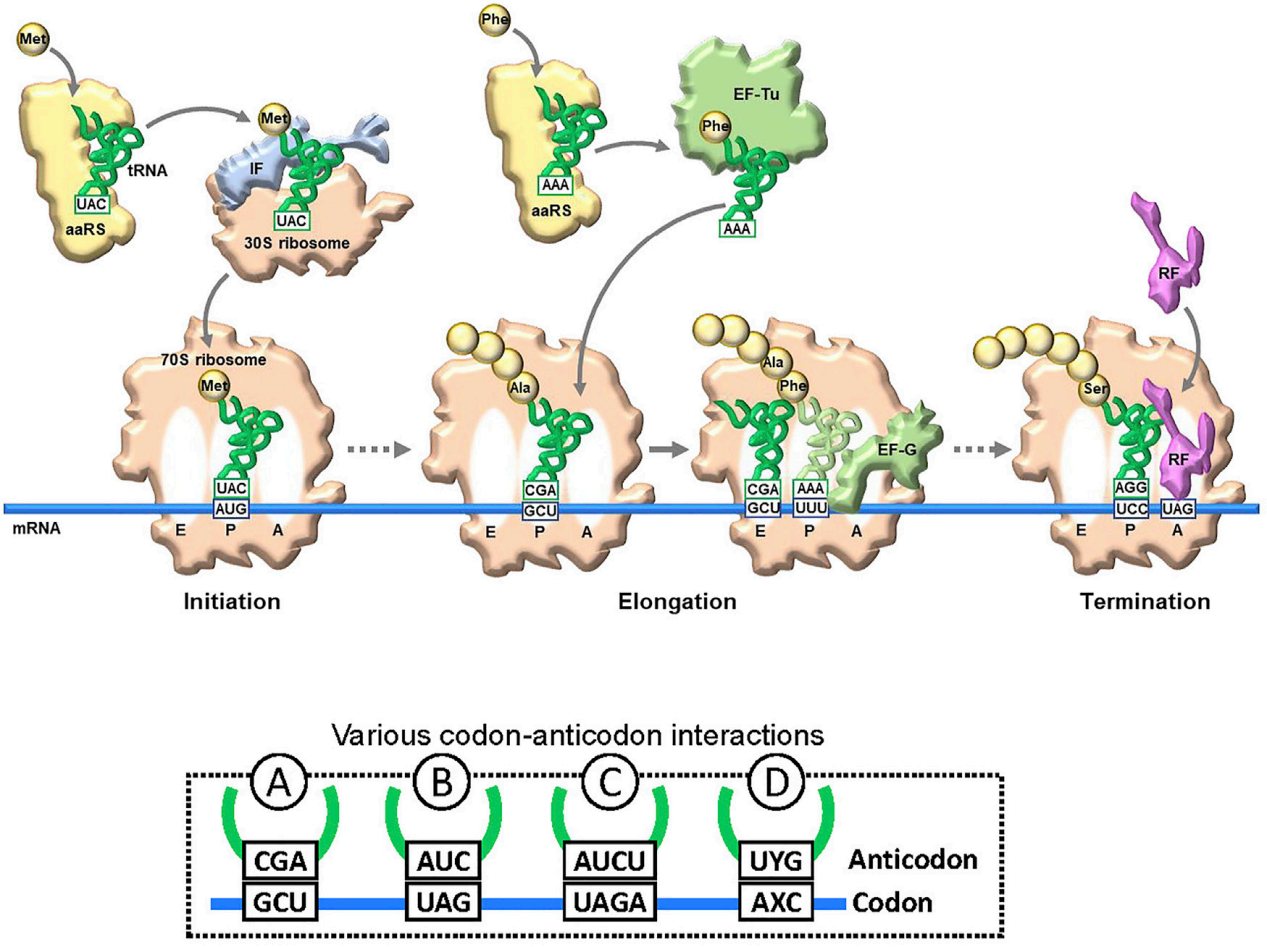
Translation and codon-anticodon interactions. Simplified illustration of translation flow from initiation to termination. The representative key components are schematically illustrated. aaRS: aminoacyl-tRNA synthetase. tRNA and mRNA are shown in blue and green lines, respectively. To decode a specific amino acid, a new codon-anticodon is required. Examples of four different codon-anticodon interactions for (re)assignment of an unnatural amino acid (unAA). A: usual natural codon; B: stop codon (UAG); C: four-base codon (quadruplet codon); D: unnatural-base codon.

Genetic code engineering based on the NBP system has a long research history. In translation, there are several checkpoints for unAA incorporation into proteins (Figure 2). A specific aminoacyl-tRNA synthetase (aaRS) is required for esterifying the unAA to a specific tRNA to generate the unAA-tRNA. Namely, an orthogonal pair of an unAA and its aaRS must be created. The unAA-tRNA should be recognized by elongation factor Tu (EF-Tu), which binds specifically to the aminoacyl-tRNA. Ribosomes must catalyze protein synthesis by incorporating the unAA, using the unAA-tRNA as a substrate. Over the past few decades, several methods to bypass these checkpoints have been developed, for genetic code expansion systems using the existing NBP system, such as the use of stop codons (Type B in Figure 2 and Figure 1C), four-base codon-anticodon interactions (Type C in Figure 2), and sense codon reprogramming (Figures 1D–F), for unAA incorporation into proteins. Importantly, these NBP methods are also employed in UBP systems, and these NBP and UBP systems could potentially be complementary to each other for the further advancement of novel translation systems involving unAA incorporation.

**FIGURE 3:**
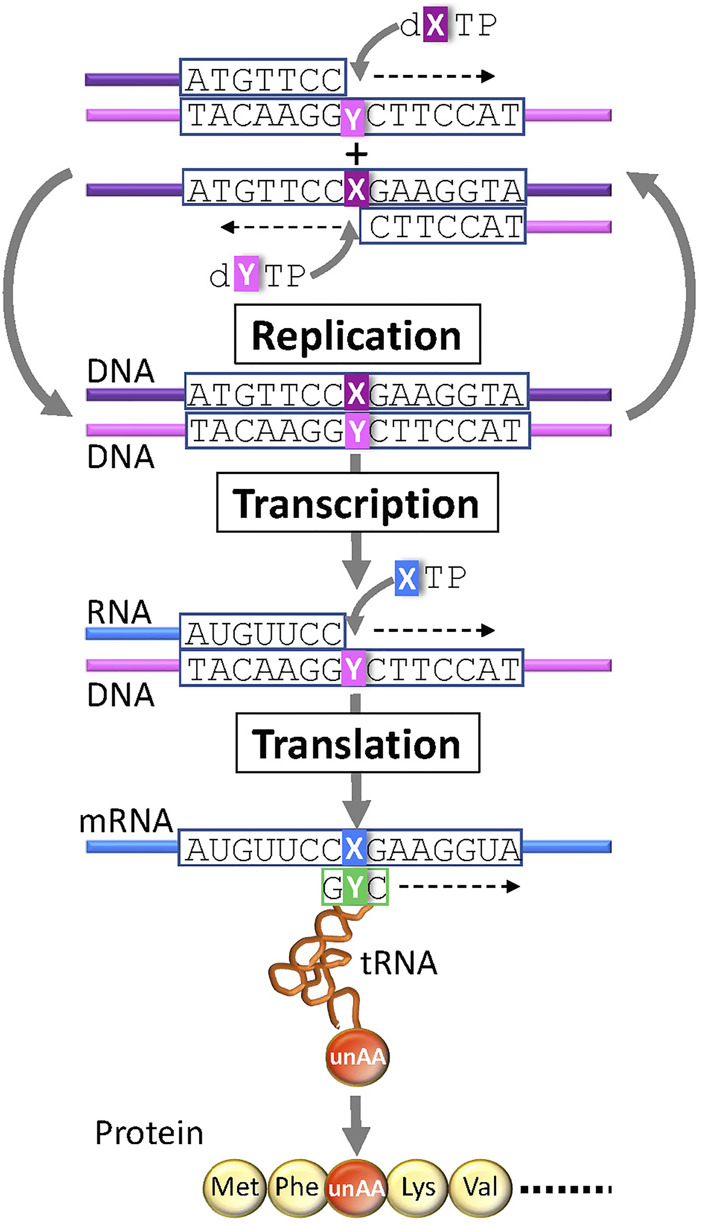
Genetic alphabet expansion using an unnatural base pair (UBP) system for genetic code expansion. A third base pair (X–Y) that functions in replication, transcription, and translation, together with the natural A–T(U) and G–C base pairs, enables the site-specific incorporation of unnatural X and Y nucleotides and unnatural amino acids (unAAs) into nucleic acids and proteins.

In the last quarter century, several UBPs that function as a third base pair in replication, transcription, and/or translation have been developed (Figure 3) (Benner et al., 2016; Kimoto and Hirao, 2020; Manandhar et al., 2021). DNAs containing UBPs are amplified and transcribed to RNA by polymerases. UBPs also create novel codon-anticodon interactions involving new letters, enabling the site-specific incorporation of unAAs into proteins by ribosome-mediated translation. Additional UBPs could largely expand the existing codon table and theoretically make 152 additional new codons [216 (= 6 × 6 × 6)—64 (= 4 × 4 × 4)] in a six-letter UB system for multiple unAA incorporations (Figure 1G and type D in Figure 2).

In this review, we will introduce the methods to create new codon-anticodon interactions for the site-specific incorporation of unAAs into proteins, using NBP and UBP systems. Basic methods for employing the NBP system to expand the codon table will be briefly mentioned. For details of the related topics, including unAA-aminoacylations of tRNAs and eukaryotic translation systems, refer to these reviews (Young and Schultz, 2010; Budisa, 2013; Lajoie et al., 2016; Agostini et al., 2017; Mukai et al., 2017; Kubyshkin et al., 2018; Melnikov and Soll, 2019; Chung et al., 2020; De la Torre and Chin, 2021). Since the UBP systems are relatively new, we will describe them in detail. Finally, we will also discuss the combination of the UBP and NBP systems and future perspectives.

## Genetic Code Expansion Using the NBP System

First, we will briefly introduce the genetic code expansion using the NBP system, which includes the use of stop codons, four-base codon-anticodon interactions, and sense codon reprogramming in prokaryotic systems.

**Use of stop codons:** The most common method for site-specific unAA incorporation is the use of stop codons. As a specific case, archaea and eukaryotes also use stop codons for the incorporation of selenocysteine and pyrrolysine into proteins, using suppressor tRNAs. There are three stop codons, amber (UAG), ochre (UAA), and opal (UGA). Among them, amber is the most popular codon for unAA incorporation (Figure 1C and type B in Figure 2), because it has the lowest frequency as a stop codon in *Escherichia coli*. The stop codon usage in *E. coli* K12 is 7% UAG, 64% UAA, and 29% UGA (Figure 1B) (Openwetware, 2012). Interestingly, the amber codon usage is also low in other organisms, but it is especially low in *E. coli* (Belin and Puigbo, 2022).

There are two methods for the unAA-aminoacylation of the suppressor tRNA with the CUA anticodon corresponding to the UAG amber codon. One is enzymatic ligation between a suppressor tRNA without the 3′-CA sequence and a chemically synthesized dinucleotide, pCA, which is aminoacylated with an unAA at the 3′-terminus (Heckler et al., 1984). This method has been used in *in vitro* translation systems for site-specific unAA incorporation into proteins (Bain et al., 1989; Noren et al., 1989). The other method is the use of a specific tRNA and aaRS pair, which can be employed in both *in vitro* and *in vivo* translation systems. Some of the tRNA-aaRS pairs are specific in each archaeon, prokaryote, and eukaryote, and tRNA-aaRS engineering studies revealed that they can potentially be used as orthogonal pairs in archaea or eukaryotes and assigned for unAAs in bacterial translation systems for unAA incorporation (Wang et al., 2001; Melnikov and Soll, 2019). For example, a tyrosyl-tRNA_CUA_ variant and its aaRS from *Methanocaldococcus jannaschii* and a pyrrolysyl-tRNA_CUA_ variant and its aaRS from *Methanosarcina barkeri* have been used as representative orthogonal pairs for unAA-tRNAs in *E. coli* and eukaryotic translation systems (Steer and Schimmel, 1999; Wang et al., 2001; Wang and Schultz, 2001; Chin et al., 2002a; Nguyen et al., 2009; Syed et al., 2019).

The issue with using stop codons is that the suppressor tRNA competes with release factors (see the left side in Figure 7A). When the ribosome reaches the stop codon on the mRNA, one of the release factors (RFs) binds to the A site for the ribosome dissociation from the mRNA (Figure 2). For example, RF1 recognizes UAG and competitively inhibits the binding of unAA-tRNA_CUA_ to UAG in mRNA, reducing the efficiency of unAA-incorporation into proteins.

To address this issue, Ueda’s team developed the PURE system (Protein synthesis Using Recombinant Element system), in which ribosomes, tRNAs, and translation factors isolated from *E. coli* are mixed for *in vitro* translation (Shimizu et al., 2001). By removing RF1 from the recombinant system, the UAG codon becomes free to encode an unAA, which increases the translation efficiency. Only UGA and UAA, which are recognized by RF2, are employed as the stop codons in the system.

Another method for *in vivo* translation was developed by removing RF1 from living organisms. Sakamoto’s team created an organism (RF1-deficient *E. coli* strain, RF_ZERO_) by replacing seven essential UAG amber codons (Mukai et al., 2010), and then further improved the strategy, by replacing 95 of the 273 UAG codons in *E. coli* with UAA or UGA stop codons (Mukai et al., 2015a). In the strain, UAG codons are used for the site-specific incorporation of unAAs. Isaacs’ team replaced all of the UAG codons at 321 positions in the *E. coli* genome with UAA, and constructed a genomically recoded organism (GRO), the C321.ΔA strain (Lajoie et al., 2013a; Lajoie et al., 2013b). Interestingly, in addition to efficient unAA incorporation into proteins, the GRO exhibited increased resistance to bacteriophage T7 infection.

**Four-base codon-anticodon interactions:** As a codon alternative, Sisido’s team developed a four-base codon system, instead of the natural three-base codon system (type C in Figure 2) (Hohsaka et al., 1996; Murakami et al., 1998). In the system, unAA-tRNAs contain four-base anticodons corresponding to the four-base codons in mRNA, and the four-base codon-anticodon interactions function in ribosome-mediated translation. The problem is that the existing tRNA_XYZ_ competes with the four-base anticodon tRNA_XYZW_ and *vice versa*. To address this issue, they first chose AGGU as the four-base codon, because the AGG codon for arginine is the least used codon in *E. coli* (AGG: 2%, AGA: 4%, CGG: 10%, CGA: 6%, CGU: 38%, and CGC: 40% for arginine) (Figure 1B). In addition, they embedded a stop codon, such as UAA, in the following frame-shifted position (i.e., AGGUCGU·AAU) (see the left side in Figure 7B). If the AGGU codon was undesirably used by tRNA_CCU_, then the translation would pause at the stop codon (i.e., AGGUCG·UAAU).

In subsequent experiments, they found that GGGU exhibits the most efficient translation efficiency among the four-base codon contexts (Hohsaka and Sisido, 2002). Using two four-base codons, AGGU and CGGG, they succeeded in the site-specific incorporations of two unAAs into streptavidin (Hohsaka et al., 1999). Hohsaka’s team achieved the site-specific labeling of proteins by using dye-conjugated amino acids as unAAs by the four-base codon system (Abe et al., 2010). Schultz’s team comprehensively examined the translation efficiency of the four-codon system and found the best four-codon contexts, AGGA, UAGA, CCCU, and CUAG (Magliery et al., 2001).

Toward *in vivo* translation systems combining the four-base codon system and the amber codon suppression, improved orthogonal pairs of aaRSs and tRNAs with four-base anticodons were developed (Magliery et al., 2001; Chatterjee et al., 2012). Chin’s team evolved a ribosome (ribo-Q1) that efficiently decodes a series of four-base and amber codons to increase the multiple incorporations of unAAs in *E. coli*. Using this ribo-Q1 system, including AGGA and UAG codons and their orthogonal tRNA-aaRS pairs, they performed the site-specific incorporation of two clickable unAA pairs, azide- and alkyne-containing amino acids, allowing for the cyclization of the generated proteins (Neumann et al., 2010). These amino acids are encoded by only one codon (Met: AUG; Trp: UGG), facilitating the replacement of these sense codons with unAAs.

**Reprogramming sense codons:** Historically, the reassignment of sense codons was first reported for unAA incorporations, in which auxotrophic bacteria were starved for one natural amino acid and supplemented with an unAA. Cowie and Cohen replaced methionine with selenomethionine, using an *E. coli* methionine auxotroph (Cowie and Cohen, 1957). Wong reported a variant of tryptophan-auxotrophic *Bacillus subtilis* using 4-fluorotryptophan, instead of tryptophan, which was created by gradually decreasing tryptophan and increasing 4-fluorotryptophan in the culture medium (Wong, 1983). Tryptophan is coded with only one codon (UGG) (Figure 1D).

As in the use of the amber codon, rare codons in a synonymous codon family for the same amino acids are useful for unAA assignment as a 21st amino acid. As mentioned above, the rare AGG arginine codon in *E. coli* was used as an unAA codon (Zeng et al., 2014). Sakamoto’s team developed an *E. coli* system to incorporate l-homoarginine into proteins, using an engineered pair of an archaeal pyrrolysyl-tRNA synthetase and tRNA^Pyl^_CCU_ for the unAA (Mukai et al., 2015b). Furthermore, they replaced AGG codons in essential genes with other synonymous Arg codons for an efficient *in vivo* unAA translation system in *E. coli*.

Another rare codon, AUA, a sense codon (Figure 1B), has also been suggested for unAA incorporation (Bohlke and Budisa, 2014). The AUA codon in *E. coli* is recognized by tRNA^Ile^ with a modified LAU anticodon (L: lysidine (2-lysyl-cytidine)), enabling L to pair with A in the codon (Suzuki and Miyauchi, 2010). Since this modification is catalyzed by TilS (Soma et al., 2003), the unmodified tRNA with CAU does not recognize the AUA codon, and thus could be used for an unAA in a TilS-depleted *E. coli* strain (Figure 1D).

The AUG codon is also a candidate for the sense codon reassignment for unAA incorporation (De Simone et al., 2016) (Figure 1D). The methionine codon AUG is used in two tRNAs: initiator tRNA^fMet^ for the initiation codon and elongator tRNA^Met^ for internal AUG codons. By eliminating the elongator tRNA^Met^ from a methionine auxotrophic *E. coli* strain, the introduction of a heterologous MetRS–tRNA^Met^ pair from the archaeon *Sulfolobus acidocaldarius* to the system allows the incorporation of methionine analogs into proteins. The initiator tRNA^fMet^ could also be used for unAA incorporation at the N-terminal position of proteins.

In *in vitro* translation systems to reassign sense codons, the PURE system is useful to specifically remove the endogenous tRNA and aaRS for each unAA. A representative method for multiple unAA incorporations is the FIT (Flexible *In-vitro* Translation) system developed by Suga’s team (Goto and Suga, 2009; Torikai and Suga, 2014). For example, they assigned GUU/C, CGU/C, and GGU/C codons to three different unAAs for the preparation of 23-letter proteins (Figure 1E) (Iwane et al., 2016). Their system also used ribozymes called Flexizymes for unAA-aminoacylation of tRNAs (Ohuchi et al., 2007; Passioura et al., 2014). Flexizymes were generated by an *in vitro* selection method using RNA libraries and an activated unAAs (Lee et al., 2000; Saito and Suga, 2001; Passioura and Suga, 2014). By applying the FIT system to ribosome display methods, they developed a platform system (RaPID, Random non-standard Peptide Integrated Discovery) to generate functional cyclic peptides from peptide libraries containing several unAAs (Passioura et al., 2014). Recently, they established a system for multiple incorporations of β-amino acids into peptides (Katoh et al., 2020), in which the tRNAs were engineered by modifying the T-stem and D-arm to increase the binding affinity to EF-Tu (Iwane et al., 2021). In this system, they used AUU/C, CAU/C, and UGU/C codons for β-amino acids, as well as AUG for unAAs, to promote the cyclization of the generated peptides. To generate stabilized inhibitor peptides, they recently reported a successful screening using a random peptide library with aromatic cyclic β^2,3^ amino acids, prepared by ribosomal incorporation (Katoh and Suga, 2022).

In the area of codon reprogramming, Chin’s team established another GRO system by the total synthesis of the *E. coli* genome with defined synonymous codon compression (Wang et al., 2016; Fredens et al., 2019). They designed and synthesized the 4-Mb *E. coli* genome, in which 18,214 codons for two serine codons (TCA and TCG) and the TAG amber codon in all of the genes were replaced with AGC, AGT, and TAA, respectively. Furthermore, the genes encoding tRNA^Ser^_UGA_, tRNA^Ser^_CGA_, and RF1 were also removed from the genome. Therefore, the synthesized *E. coli* (Syn61) uses 61 codons, and the three vacant codons can be used for three unAA codons (Figure 1F).

## Genetic Code Expansion Using UBP Systems

**FIGURE 4:**
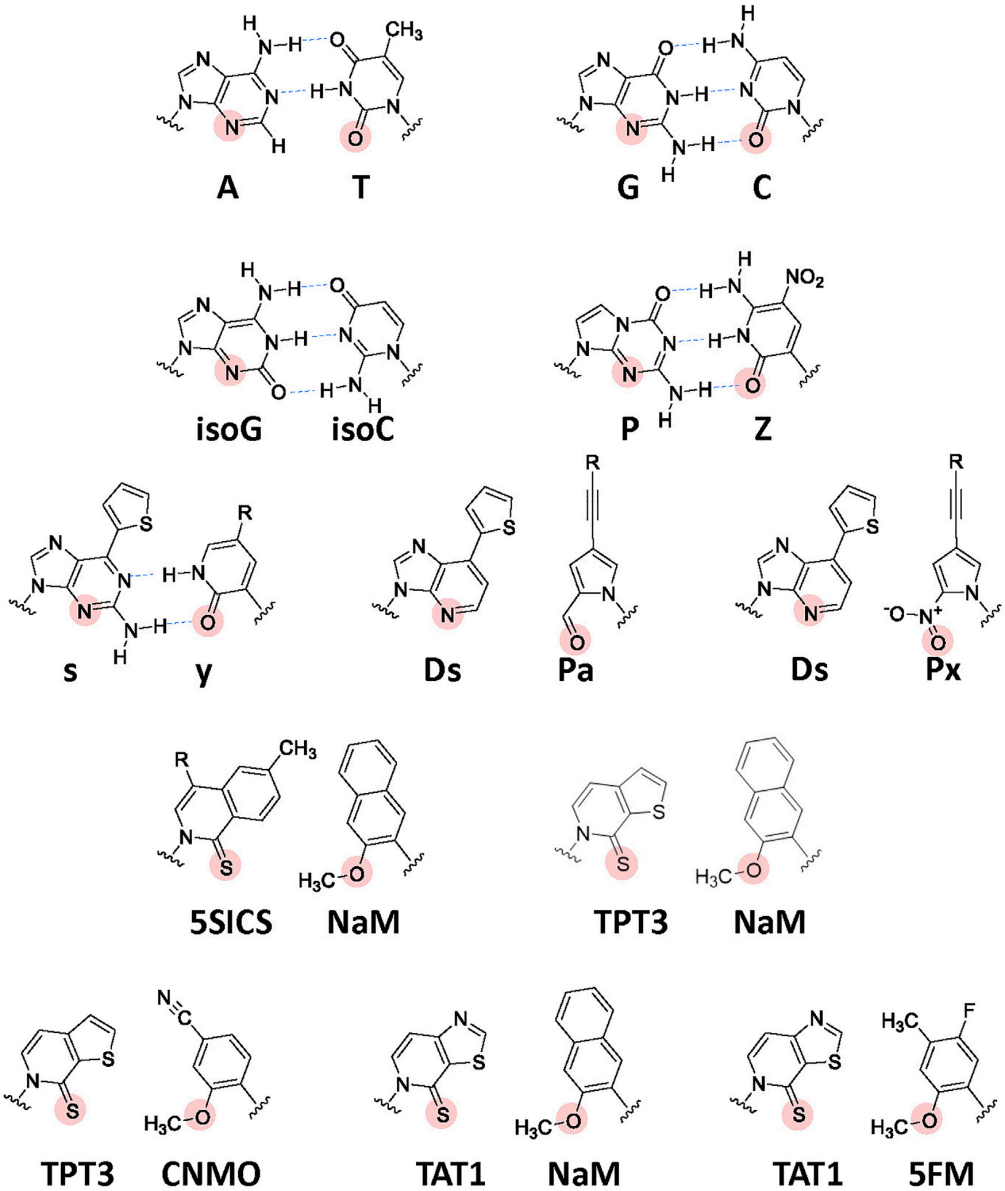
Chemical structures of the natural Watson–Crick base pairs and a variety of UBPs developed to expand the genetic alphabet. Hydrogen-bonding interactions between the cognate base pairs are shown by blue arrows. The important residues (hydrogen acceptors) recognized by DNA and RNA polymerases are indicated by solid circles. R: further modification is available by attaching a variety of functional groups *via* linkers.

**Development of UBP systems *in vitro*:** In 1962, Alexander Rich proposed the potential use of a UBP, isoguanine (isoG) and isocytosine (isoC) (Figure 4) with different hydrogen-bonding patterns from those of G–C, for a new codon-anticodon system (Rich, 1962). Even though the codon table was still being deciphered at that time, he imagined that two-base genetic codons, instead of three-base codons, using six-letter genetic alphabets could cover the 20 standard amino acids (20 < 6 × 6). Over 2 decades later, in the late 1980s, Benner’s team designed several UBPs with alternative hydrogen bonding patterns, including the isoG–isoC pair, and chemically synthesized these UB units. Their biological results opened a new world in which the UBPs could be used for replication and transcription, with orthogonal base pairing to the two natural base pairs (Switzer et al., 1989; Piccirilli et al., 1990). In 1992, they reported an *in vitro* translation system for the site-specific incorporation of 3-iodotyrosine into a peptide, using chemically synthesized mRNA with an (isoC)AG codon and tRNA with a CU(isoG) anticodon (Figure 1G) (Bain et al., 1992). Their efforts toward further UBP development and optimization led to the replicable and transcribable P–Z pair, with higher fidelity than those of the isoG–isoC pair (Figure 4) (Yang et al., 2010; Yang et al., 2011).

In the late 1990s, Romesberg’s and Hirao’s teams also started to develop UBPs, based on different concepts, toward practical applications to increase the functionalities of nucleic acids and proteins beyond the canonical four-letter biological system. More than 20 years on, several representative UBPs have become applicable in replication, transcription, or translation *in vitro* and/or *in vivo*, including the s–y and Ds–Pa/Ds–Px pairs from Hirao’s team and the NaM–5SICS/NaM–TPT3/CNMO–TPT3/NaM–TAT1 pairs from Romesberg’s team (Figure 4) (Hirao et al., 2002; Hirao and Kimoto, 2012; Malyshev and Romesberg, 2015; Kimoto and Hirao, 2020; Manandhar et al., 2021).

Hirao’s team developed the s–y pair, which functions as a third base pair in transcription (Figure 4). The bulky thienyl group in the s base eliminates its pairing with the natural bases, and the y substrate (yTP) is site-specifically incorporated into RNA opposite s in the DNA template by T7 RNA polymerase (Fujiwara et al., 2001; Hirao et al., 2002). The s–y pair was applied to an *in vitro* transcription-translation system for the incorporation of unAAs into a specific position of the 185-aa Ras protein. Using an 863-mer DNA template containing s and 3-chlorotyrosyl-tRNA_CUs_ (ClTyr-tRNA_CUs_), they coupled the T7 transcription with *in vitro* translation using an *E. coli* cell-free system. The LC-MS analysis of the obtained protein confirmed that the yAG codon in the transcribed *ras* mRNA was decoded by the CUs anticodon of ClTyr-tRNA_CUs_ (Figure 1G). Although the yAG codon was decoded by the native Lys-tRNA_UUU_ and Gln-tRNA_CUG_ in the absence of ClTyr-tRNA_CUs_, the undesired misincorporation was competitively suppressed by the predominant ClTyr incorporation by ClTyr-tRNA_CUs_ (Hirao et al., 2002). Even though the yAG codon is closely related to the UAG amber codon, the translation experiments without ClTyr-tRNA_CUs_ revealed that the replacement of one of the bases in the termination codons with an unnatural base bypasses the competition with release factors (see Figure 7A).

In the translation system, tRNA_CUs_ was prepared by ligation of the 5′-half fragment derived from the native *Saccharomyces cerevisiae* tRNA^Tyr^ with the chemically synthesized 3′-half fragment containing CUs (Ohtsuki et al., 1996). The aminoacylation of tRNA_CUs_ with ClTyr was performed by *S. cerevisiae* tyrosyl-tRNA synthetase, which does not recognize the third anticodon position (Chow and Rajbhandary, 1993; Tsunoda et al., 2007), the s position, and aminoacylates *S. cerevisiae* tRNA^Tyr^ with tyrosine and tyrosine analogs, such as 3-halotyrosine and DOPA, under specific conditions in the presence of 20% dimethyl sulfoxide and 0.25% Tween-20. In addition, the *S. cerevisiae* tRNA^Tyr^ is not aminoacylated by *E. coli* tRNA synthetase.

Hirao’s team subsequently developed the hydrophobic Ds–Pa/Px pairs, which exhibit high fidelity in replication and transcription, by removing the hydrogen-bonding interactions between pairing bases (Hirao et al., 2006; Hirao et al., 2007; Kimoto et al., 2009; Yamashige et al., 2012). The Ds–Px pair was applied to high-affinity DNA aptamer generation, thus demonstrating how unnatural components greatly increase nucleic acid functionalities (Kimoto et al., 2013; Matsunaga et al., 2017; Futami et al., 2019; Matsunaga et al., 2021).

**FIGURE 5:**
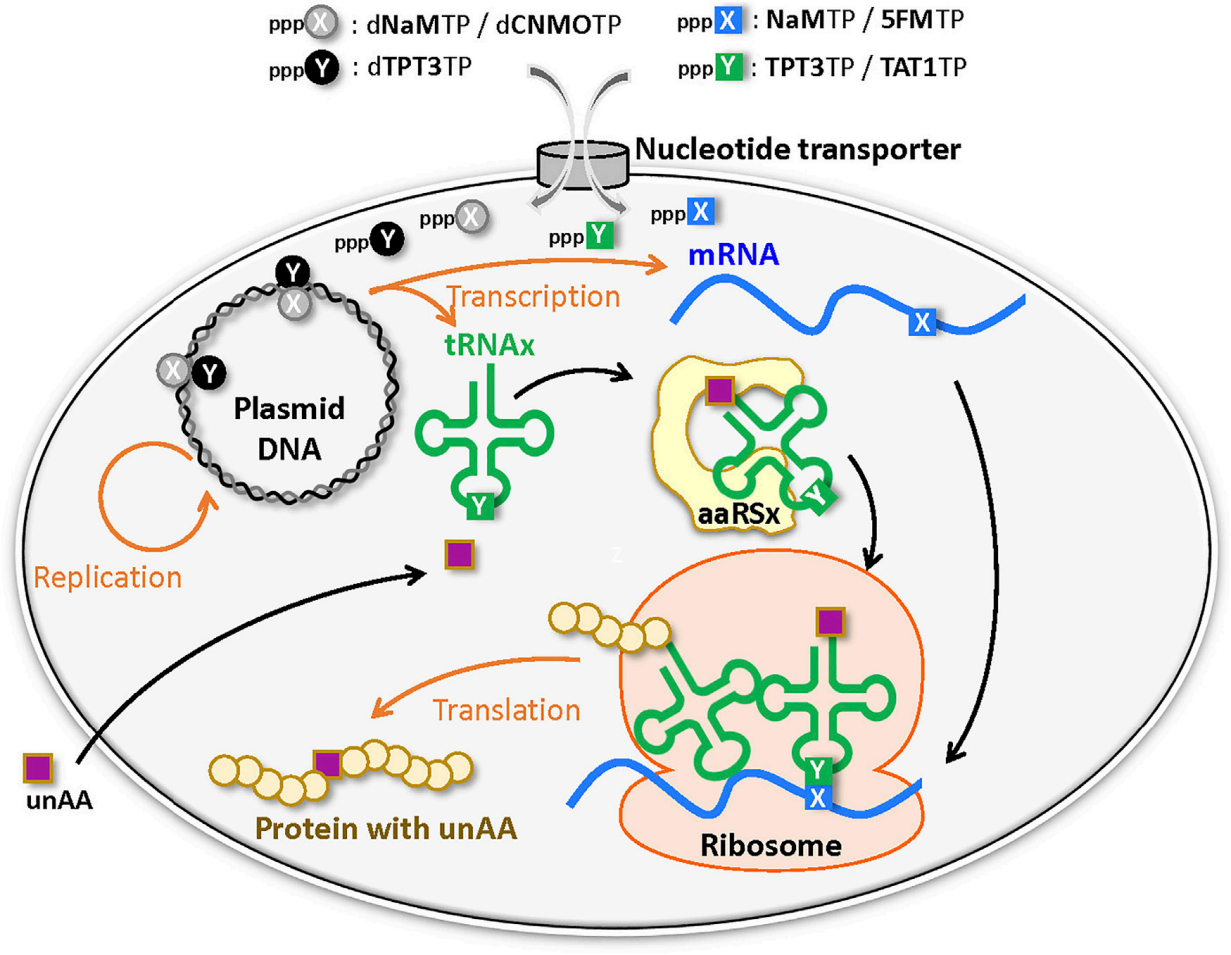
Semi-synthetic organism (SSO) that stores and retrieves the six-letter genetic information expanded by an unnatural base pair (X–Y) system.

**Development of UBP systems *in vivo*:** Romesberg’s team also developed a series of hydrophobic UBPs, such as NaM–5SCIS and NaM–TPT3 (Figure 4), with high fidelity in replication and transcription (Malyshev et al., 2009; Seo et al., 2009; Seo et al., 2011; Malyshev et al., 2012; Lavergne et al., 2013). In 2014, using the NaM–5SCIS/TPT3 pairs, Romesberg’s team successfully created an engineered *E. coli* strain (Figure 5) (Semi-synthetic organism, SSO), which replicates six-letter DNA, using their NaM–5SCIS/TPT3 pairs (Malyshev et al., 2014). To supply the UB substrates for the living cells, they employed media supplemented with UB-nucleoside triphosphates, dNaMTP and d5SCISTP. To facilitate the import of sufficient amounts of the UB substrates within the cells, the nucleoside triphosphate transporter from *Phaeodactylum tricornutum* (*Pt*NTT2) was expressed in *E. coli* [C41 (DE3) strain]. They prepared a plasmid DNA containing the NaM–TPT3 pair through PCR amplification and transformed it into the engineered *E. coli* expressing *Pt*NTT2. An analysis of the cultured cells revealed the successful replication of the six-letter plasmid DNA with reasonable retention of the NaM–5SICS pair. These results also demonstrated that the UBP was not extensively rejected as a foreign component by the repair system.

They further improved this first generation SSO by switching from the original *E. coli* strain to the BL21 (DE3) strain, which is more suitable for protein expression. The *Pt*NTT2 expression was also optimized because the extremely high expression using T7 RNA polymerase inhibited cell growth. They modified *Pt*NTT2 expression by 1) removing the N-terminal signal peptide (65 aa) of *Pt*NTT2, which is toxic to cell growth, 2) using the optimized codon usage, 3) choosing the best RNA polymerase II promoter sequence, and 4) encoding the truncated *Pt*NTT2 gene within a *lacZYA* locus in the genome, to avoid expression plasmid copy number variations. The resultant second-generation SSO, called the YZ3 strain, greatly increased the retention rates of their UBPs in replicated DNA within various sequence contexts (Zhang et al., 2017a).

In 2017, their team reported successful *in vivo* transcription and translation using the YZ3 strain, to decode the AXC or GXC codon (X = NaM) by the corresponding GYU or GYC anticodon (Y = TPT3), thus enabling the site-specific incorporation of unAAs into a recombinant superfolder green fluorescent protein (sfGFP) (Figures 1G, 4) (Zhang et al., 2017b). The YZ3 cells carrying a specific aaRS expression plasmid were additionally transformed by the plasmid DNA containing the NaM–TPT3 pairs, encoding mRNA (X) and tRNA (Y). The induced T7 RNA polymerase expression in the SSO allowed successful T7 transcription of UB-containing mRNA and tRNA, and finally yielded the GFP with an unAA at the AXC or GXC codon position, decoded by unAA-tRNA_GYU_ or unAA-tRNA_GYC_.

To evaluate the decoding of the new codon-anticodon interactions involving the NaM–TPT3 pair, they first investigated the incorporation of serine into GFP at position 151 (the TAC codon was replaced by the unnatural codon AXC) through *E. coli* tRNA^Ser^ (*ser*T), where the anticodon was replaced by the unnatural codon GYT. This system can eliminate complicated situations related to unAAs because *E. coli* serine aaRS does not recognize the anticodon for tRNA amino acylation (Shimizu et al., 1992). The efficient full-length sfGFP production with 98.5 ± 0.7% incorporation of serine at position 151 was confirmed in the cells transformed with the plasmid encoding both sfGFP(AXC)^151^ and tRNA^Ser^_GYT_, cultured in the presence of deoxy- and ribo-nucleoside triphosphates (dNaMTP, dTPT3TP, NaMTP, and TPT3TP). After the validation of the unnatural codon-anticodon interactions, they focused on unAA *N* ^6^-[(2-propynyloxy)carbonyl]-l-lysine (PrK) incorporation in sfGFP(AXC)^151^ or sfGFP(GXC)^151^, utilizing a pair of the *Methanosarcina mazei* tRNA^Pyl^_GYU_ or RNA^Pyl^_GYC_ and the *Methanosarcina barkei* pyrrolysyl-tRNA synthetase (PylRS) (Nguyen et al., 2009; Chatterjee et al., 2013). The PylRS was encoded in a separate plasmid with expression controlled by IPTG induction. For another unAA p-azido-phenylalanine (*p*AzF) in sfGFP(AXC)^151^, they utilized an evolved *Methanococcus jannaschii* TyrRS/tRNA^Tyr^ pair (i.e. *p*AzFRS.tRNA^*p*AzF^_GYU_) (Chin et al., 2002b).

By assessing the UBP retention in plasmid DNAs, the incorporation efficiencies of unAAs, and the cell growth, Romesberg’s team has further optimized the second-generation SSO. First, they examined the *in vivo* replication mechanisms for UBPs and found that the elimination of RecA and the release of DNA Pol II from SOS repression increased the UBP retention. This study resulted in the third generation SSO with an error-avoidance mechanism, called the ML2 strain [BL21 (DE3) *lacZYA::PtNTT2(66*-*575) ΔrecA polB* ^++^] (Ledbetter et al., 2018). Next, they explored a variety of UB substrate analogs for DNA replication and RNA transcription *in vivo.* They identified the CNMO–TPT3 pair, which is superior to the NaM–TPT3 pair for efficient *in vivo* DNA replication, and the 5FM–TPT3 and NaM–TAT1 pairs, which are better for the efficient production of GFP with an unAA. The optimized SSO with the dCNMOTP–dTPT3TP/NaMTP–TAT1TP system efficiently produced the GFP with three proximal unAAs, using the AXC-GYU codon-anticodon interaction (Feldman et al., 2019).

Using the ML2 strain, Romesberg’s team further identified new codons for efficient production of proteins with unAAs. In 2021, they reported that twenty UB codons are available: seven of the NXN-NYN codon-anticodon interactions (X = NaM, Y = TPT3; including UXC-GYA, CXC-GYG, AXC-GYU, GXU-AYC, GXC-GYC in clonal SSOs) and thirteen of the NNX-XNN interactions (X = NaM; including UUX-XAA, UGX-XGA, CGX-XCG, AGX-XCU in clonal SSOs) (Figure 1G) (Fischer et al., 2020; Romesberg, 2021). Interestingly, only NaM, and not TPT3, is acceptable for the codon in the second and third positions, and the third position should be the self NaM–NaM pair, rather than the hetero NaM–TPT3 pair. In addition, they confirmed that at least three of the codon-anticodon interactions, AXC-GYU, GXC-GYC, and AGX-XCU, are orthogonal to each other, allowing for simultaneous decoding in the SSO (Fischer et al., 2020). By measuring the transcription fidelity *in vivo*, they found that the decoding at the ribosome is more sensitive than transcription (Zhou et al., 2020). This might be because the variable codon performance is the total output of the sequence-dependent translation efficiency, although the recognition of the codon-anticodon interaction might differ in eukaryotic cells (Zhou et al., 2019). They are currently exploring the recognition of the NaM–TPT3 pair by the multi-subunit *E. coli* RNA polymerase II, as compared to the single-subunit T7 RNA polymerase, to create next generation SSOs (Hashimoto et al., 2021; Oh et al., 2021).

**FIGURE 6:**
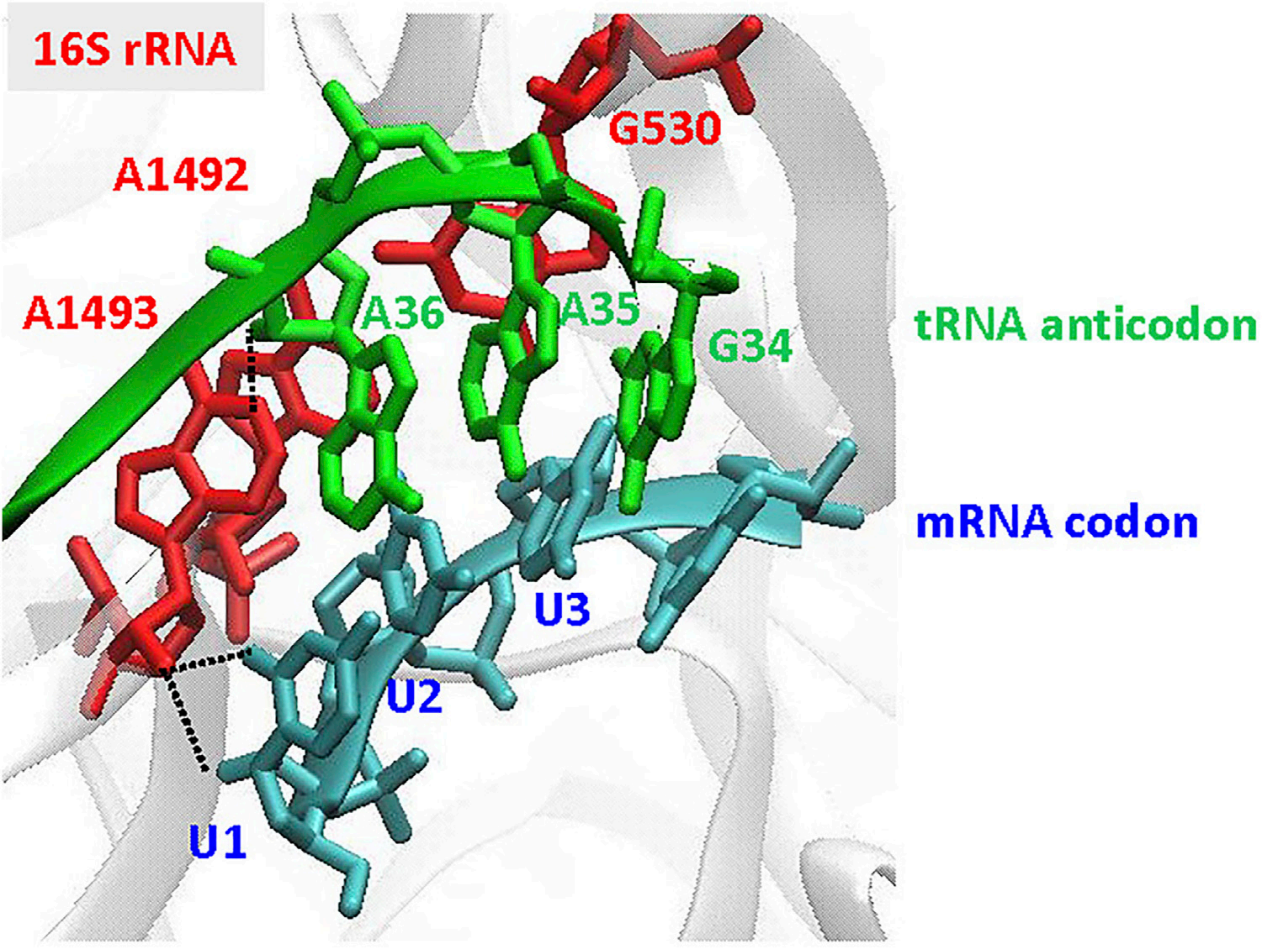
Recognition of the codon-anticodon interaction at the ribosome. The illustration is focused on the decoding site of the 30S subunit, showing the A-site codon (UUU in blue) and the tRNA anticodon (GAA in green), using the coordinates in PDB: 1IBM (Ogle et al., 2001). The important nucleotides, G530, A1492, and A1493, in 16S rRNA are indicated in red. Type I A-minor motif interactions, found in A1493 at the first codon-anticodon position (U1 A36), are indicated by black dotted lines.

**Recognition of the codon-anticodon interaction in the bacterial ribosome:** For accurate discrimination between cognate and near- or non-cognate aa-tRNAs, the three highly conserved G530, A1492, and A1493 bases in 16S rRNA are prerequisite (Figure 6). To select the cognate tRNA through the minor groove interactions of the codon-anticodon interaction, the two adenine bases are flipped out from the internal loop of helix 44 of 16S RNA in the 30S ribosomal subunit (Ogle et al., 2001; Ramakrishnan, 2002; Cochella et al., 2007). At the first position of the codon-anticodon, A1493 forms a type I A-minor motif interactionboth the O2’ and N3 of A1493 are located in the minor groove of the first position, maximizing the number of hydrogen bonds that can be formed (Nissen et al., 2001; Ogle et al., 2001). At the second position, A1492 and G530 are tightly packed in the minor groove of the codon-anticodon (a type II A-minor motif interaction) (Nissen et al., 2001; Ogle et al., 2001), but do not directly interact with the base moieties. Thus, the second position would accommodate small structural differences in the base pair (Fischer et al., 2020). The third position has more open space and is less monitored by the ribosome.

Recent *in vitro* translation studies using RNA nucleobase derivatives in the mRNA demonstrated that the hydrogen-bonding interaction between the N1 of purines and the N3 of pyrimidines is sufficient for decoding at the first or second position. At the third “wobble” position, an adequate stacking force, not limited to the hydrogen-bonding interactions, could be essential (Hoernes et al., 2018). The UBP studies clearly demonstrated that the acceptance of the hydrogen-bonded isoC-isoG and y–s pairs at the first codon-anticodon position might be reasonable, since their UB pairing structures effectively mimic the natural Watson-Crick base pairing geometry. In contrast, the non-hydrogen-bonded NaM–TPT3 pair at the first position might adopt a cross-strand intercalated structure (Manandhar et al., 2021), as found in the free DNA duplex form. The UBP is quite different from the Watson-Crick like structure found in the polymerase active site (Betz et al., 2012; Betz et al., 2013), which might prevent recognition as a cognate base pair. Interestingly, although the previous UBP study suggested that at least the hydrogen-bonding interaction between the N1 of purines and the N3 of pyrimidines at the second position is required for the decoding, the NaM–TPT3 pair is well accepted as cognate. Together with the acceptance of the self NaM–NaM pair at the third position, these results indicate that complementary packing and hydrophobic forces can “bypass” the requirement for precise decoding at the second and third positions (Hoernes et al., 2018). However, the reason why only NaM, and not TPT3, is acceptable for mRNA remains unclear.

The discrimination mechanisms of cognate and non-cognate pairs by DNA and RNA polymerases may be similar to those of the decoding process. Both polymerases and the ribosome 30S subunit undergo a structural rearrangement from an open to a closed form in the cognate pairing, through interactions with the minor groove of the Watson-Crick base pairing (Ogle et al., 2002). Further detailed translation studies using other UBPs might elucidate the unknown mechanisms and driving forces by which the RNA-based decoding system precisely discriminates the cognate and non-cognate pairing, commonly and/or differently from those in polymerases.

## Therapeutic Applications of Engineered Proteins by Genetic Code Expansion

These translation systems by genetic code expansion have facilitated the rational design and optimization of proteins suitable for therapeutic applications, by improving the biological functions and pharmacokinetics of biologics in a manner resembling the pursuit of small-molecule therapeutics. Several macrocyclic peptides containing unAAs, including those generated by the ribosomal translation system, are now undergoing clinical tests (Vinogradov et al., 2019). Currently, several engineered proteins generated by these technologies are in pre-clinical and clinical trials as protein therapeutics. Representatives are, but not limited to, PEGylated interleukin-2 (SAR44425, THOR-707) (Manandhar et al., 2021), PEGylated fibroblast growth factor 21 (BMS-986036, pegbelfermin), Anti-HER2 antibody-drug conjugate (a site-specific Herceptin-monomethyl auristatin D (MMAD) conjugate, ARX788) (Sun et al., 2014), and anti-CD3 Folate Bi-Specific (Sun et al., 2014).

The recombinant human cytokine interleukin-2 (rhIL-2, or aldesleukin) was originally approved as a drug for immune oncology targeting renal cell carcinoma (Klapper et al., 2008; Krieg et al., 2010). However, rhIL-2/aldesleukin therapy, targeting the stimulation of tumor immune responses through CD8^+^ effector T and natural-killer cells, which express the IL-2 receptor beta and gamma subunit complex (IL-2 Rβγ), has been limited due to the short half-life and off-target effects resulting from its interaction with the IL-2 receptor alpha subunit (IL-2 Rα). Scientists at Synthorx (founded by Romesberg in 2014, acquired by Sanofi in 2019) used Romesberg’s SSO (YZ3 strain) to successfully identify a suitable PEGylated position (P65) in IL-2 from 10 candidates (K35, R38, T41, F42, K43, Y45, F62, P65, E68, and V69) and addressed the above two issues. These analyses resulted in the development of THOR-707, the IL-2 compound with an unAA at position 65, followed by further modification with a 30 kDa mPEG, which retained the binding ability to IL-2 Rβγ but lacked that to the undesired IL-2 Rαβγ, and showed an extended half-life (Manandhar et al., 2021; Ptacin et al., 2021; Romesberg, 2021). THOR-707 is currently in a phase I/II study, not only as a monotherapeutic, but also in combination with a checkpoint inhibitor (pembrolizumab or cemiplimab) (Romesberg, 2021).

## Conclusion and Future Perspective

**FIGURE 7:**
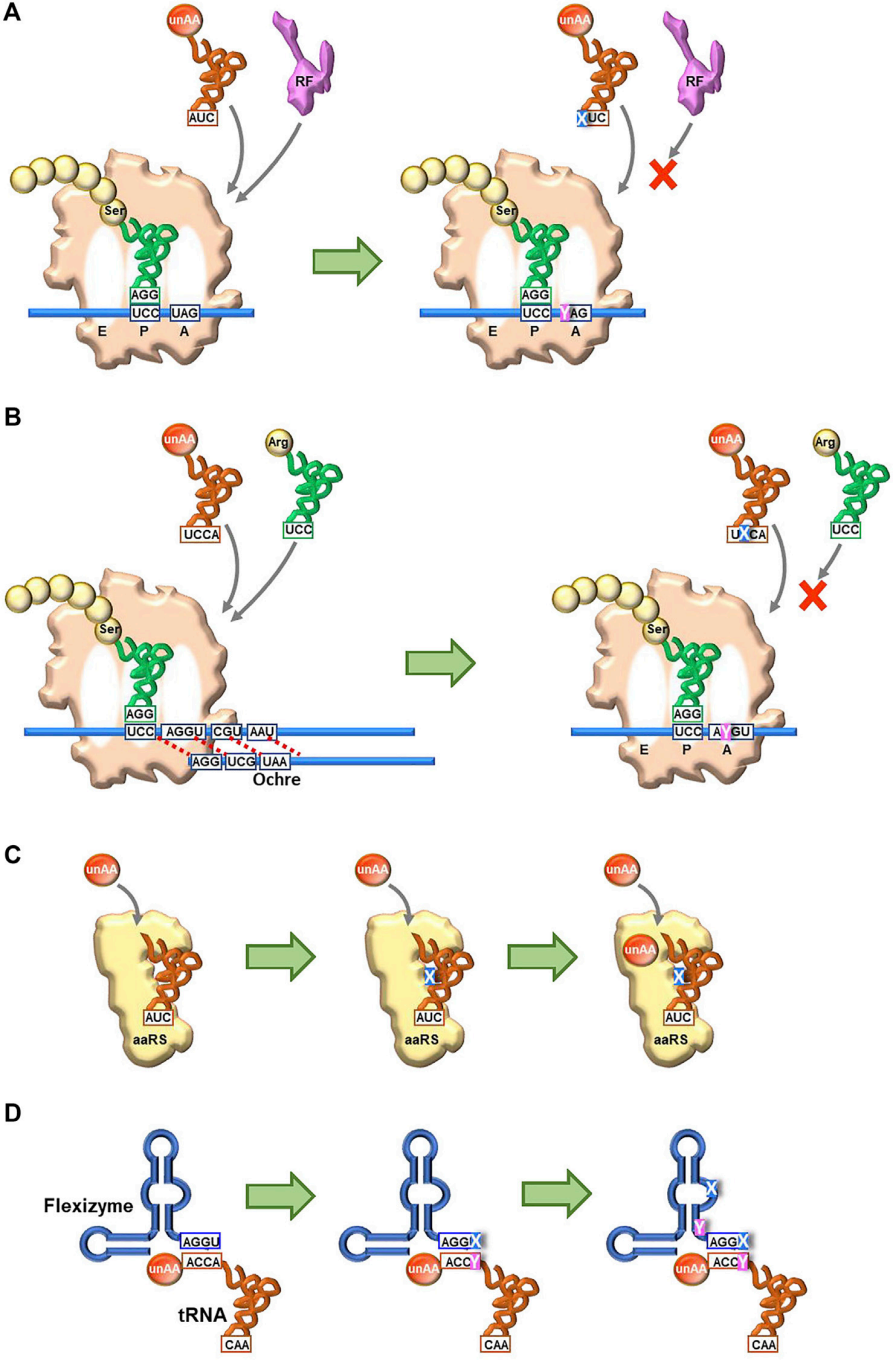
Future perspectives to create new-anticodon interactions using UBP systems for unAA incorporation **(A)** Use of a stop codon (UAG) **(B)** Usage of a four-base codon (quadruplet codon) **(C)** Creation of new orthogonal engineered tRNA and aaRS pairs for specific unAA aminoacylation, using unnatural nucleotides and unAAs **(D)** Creation of new aminoacylation systems using engineered Flexizymes containing unnatural nucleotides.

Continuous and comprehensive research on genetic alphabet rearrangement and expansion technologies has largely improved the unAA incorporation fidelity and efficiency and created new organisms. Even hydrophobic UBPs without any clear hydrogen-bond interactions between pairing bases can function as new letters of DNA and RNA, for information storage and retrieval in *in vivo* systems (SSOs). Recent breakthroughs in UBP development as a third base pair have created novel genetic alphabet systems of DNA and RNA, providing the expanded codon table in translation, which can bypass the checkpoints of the native translation system. The new UB-codons related to the stop codons, such as isoCAG, yAG, and UGNaM, predominantly interact with their UB-anticodons and prevent the interaction with RF (Figure 7A.

The UBP systems could improve the current NBP genetic code expansion systems. For example, the introduction of UBs into four-base codon systems might prevent the competition with the native tRNAs with three-base anticodons (Figure 7B). As shown in Romesberg’s results, embedding the UB in the middle of a codon (for example, AXC and GXC) would maximize the UB’s discrimination capabilities in decoding. A novel pair of tRNA and aaRS for unAAs could be created by introducing UBs into tRNAs and unAAs into aaRSs (Figure 7C) (Young and Schultz, 2018), as UB-containing nucleic acid aptamers significantly increase the affinities and specificities with target proteins (Kimoto et al., 2013). Such novel pairs would enhance the simultaneous incorporation of different multiple unAAs, as well as the known tRNA/aaRS pairs (Chin et al., 2003; Brustad et al., 2008; Tanrikulu et al., 2009; Italia et al., 2017; Melnikov and Soll, 2019; Ding et al., 2020). Flexizymes could also improve the efficiency and specificity of tRNA aminoacylation by introducing UBs, although the UBP applications to ribozymes have not yet been reported. The current Flexizymes recognize tRNAs by the interaction between the terminal GGU sequence of Flexizyme and the terminal ACCA sequence of tRNAs, and thus aminoacylate tRNAs non-specifically (Figure 7D). One possible improvement would be the introduction of a UBP (X-Y) to the terminal position (GGX) of Flexizyme and to the discriminator base (YCC) in tRNAs. Further research for the introduction of UBs (UBPs) and unAAs into biopolymers (DNA, RNA, and protein) would yield not only fruitful findings in translation mechanisms but also novel protein therapeutics, empowered by the cooperative fusion of chemistry and biology. Furthermore, the UBP-unAA systems have the potential to create novel organisms with increased functionalities, such as enhanced productivity of useful materials and highly sensitive sensors for detection. Thus, the combination of the NBP and UBP systems could further expand the capability of genetic code engineering for multiple unAA incorporations.

Currently, only the UBPs developed by Romesberg’s team have been demonstrated in the *in vivo* system. The potentials of other UBPs, such as Z–P and Ds–Px, for *in vivo* systems are still unknown. The fidelity and toxicity of UBPs and UB materials and the limitation of the number of UB-codons available in SSOs are important issues. Romesberg’s team demonstrated that the use of a Cas9-based editing system allowed the increased retention (fidelity in replication) of their UBPs in living cells (Zhang et al., 2017a), but the additionally expressed sgRNAs might interfere with efficient translation. Although *in vitro* studies revealed that the replication fidelities of some UBPs are more than 99.8% per duplication, there is still room for further improvement of the specificity and stability of UBPs to reduce the mutation rates in replication, transcription, and translation. The toxicity of continuously supplementing unnatural base substrates as a third base pair for long term cultures and the possible increase in mutations have not been fully elucidated. The current UBP translation systems have mainly been studied in prokaryotic systems. In the future, UBP studies will be expanded to eukaryotic systems (Zhou et al., 2019) and provide further information and possibilities.

UBP research and its applications have only just begun. Nevertheless, the improvements of UBP systems have opened the door to novel biotechnologies, as described here. Replication with non-hydrogen bonded UBPs in the *E. coli* genome has also become an achievable target (Ledbetter et al., 2018). However, as compared to the NBP system, the utility of the current UBP systems is still limited due to the comparatively lower fidelity and efficiency with some sequence biases, which increase the mutation rates and change the evolutionary equilibrium of the system and SSOs. As for the codon-anticodon interactions, non-hydrogen bonded UBPs are less stable, but can be used in translation within the limits of the codon usage. Extensive studies using UBPs and modified nucleotides, including the further development of UBPs and the combination with NBP systems, might reveal the unknown secrets of the current life system.
